# MMP-1, UCH-L1, and 20S Proteasome as Potential Biomarkers Supporting the Diagnosis of Brain Glioma

**DOI:** 10.3390/biom12101477

**Published:** 2022-10-13

**Authors:** Lukasz Oldak, Sylwia Chludzinska-Kasperuk, Patrycja Milewska, Kamil Grubczak, Joanna Reszec, Ewa Gorodkiewicz

**Affiliations:** 1 Bioanalysis Laboratory, Faculty of Chemistry, University of Bialystok, Ciolkowskiego 1K, 15-245 Bialystok, Poland; 2 Doctoral School of Exact and Natural Science, Faculty of Chemistry, University of Bialystok, Ciolkowskiego 1K, 15-245 Bialystok, Poland; 3 Biobank, Biobank at Medical University of Bialystok, Waszyngtona 13, 15-269 Bialystok, Poland; 4 Department of Regenerative Medicine and Immune Regulation, Medical Univeristy of Bialystok, Waszyngtona 13, 15-269 Bialystok, Poland; 5 Department of Medical Pathology, Medical University of Bialystok, Waszyngtona 13, 15-269 Bialystok, Poland

**Keywords:** glioma, liquid biopsy, SPRi biosensor, biomedical research

## Abstract

The diagnosis of brain gliomas is mainly based on imaging methods. The gold standard in this area is MRI. Recommendations for the prevention, diagnosis, and treatment of gliomas are periodically modified and updated. One of the diagnostic techniques used when a brain glioma is suspected is liquid biopsy. However, this technique requires further development to confirm its effectiveness. This paper presents a proposal of three potential biomarkers of brain gliomas—extracellular matrix metalloproteinase-1 (MMP-1), ubiquitin carboxy-terminal hydrolase L1 (UCH-L1), and the 20S proteasome—which were quantified in blood plasma using SPRi biosensors. A statistical analysis of the results indicated no significant changes in the concentrations between the control group (K) and grades G1 and G2, and similarly between grades G3 and G4. However, the differences in the concentrations between the groups K/G1/G2 and G3/G4 were statistically significant. A positive average correlation was found between the concentrations of the proteins and the patient’s age. The individual tested proteins were also highly correlated with each other. Our work proposes a new diagnostic technique that may aid in the diagnosis of brain gliomas.

## 1. Introduction

The recommendations for the prevention, diagnosis, and treatment of brain gliomas have recently been updated. The diagnostic part of the update on which this publication focuses is based on the updated Central Nervous System Tumors Classification proposed by the WHO in 2016 and the latest EANO recommendations for the diagnosis and treatment of diffuse gliomas in adult patients [1]. The table below (Table 1) summarizes the preoperative diagnostic methods and techniques used when a cerebral glioma is suspected.

Therapeutic decisions are made on the basis of a histological analysis of the brain tissue. The sample should be taken in such a way as to be as representative as possible. Unless there are medical contraindications, the most common method is a stereotaxic biopsy, which involves taking a sample of the affected tumor mass with a suitable needle.

**Figure 1:**
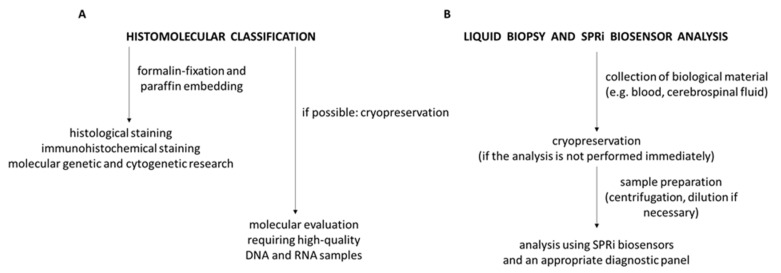
Scheme of procedure for histomolecular classification (**A**) and liquid biopsy (**B**). Diagram based on [1].

Biomarkers such as the IDH mutation, 1p/19q coding, and MGMT promoter methylation are homogeneously present in brain tumors. However, there is also a group of biomarkers whose levels depend on the place of occurrence. It is recommended in such cases to collect samples from various areas of the tumor, using both stereotactic and open-biopsy methods [1]. Figure 1 shows a diagram of the procedure for histomolecular examinations and liquid biopsy.

The diagnosis of a glioma begins with tests using magnetic resonance imaging (MRI). However, MRI is not sufficient to reliably differentiate non-neoplastic lesions and glial tumor subtypes; therefore, tissue biopsy is an integral part of the diagnosis, which enables the determination of tissue markers. For the determination of tissue biomarkers, it is necessary to perform a biopsy using a surgical procedure that is costly and very invasive for the patient. It also becomes unfeasible when the patient’s condition significantly worsens or when the neoplastic tumor is so positioned that access to its tissue is very difficult. Gliomas are heterogeneous; therefore, it has been suggested that markers circulating in the blood may better represent the cancer cell population than tissue biopsy [5]. Therefore, it is important to discover new molecular markers that will be present not only in the tumor tissue, but also in the peripheral blood, which can be tested using liquid biopsy methods. It allows for the quantitative determination of biomarkers based on a sample of peripheral blood. The proposed methods based on SPRi biosensors are in line with the current research trend. The biomarkers presented in this paper offer to distinguish milder grades of glioblastomas from advanced ones, using only blood plasma. The proposed solutions make it possible to diagnose and differentiate low- and high-grade gliomas.

**Figure 2:**
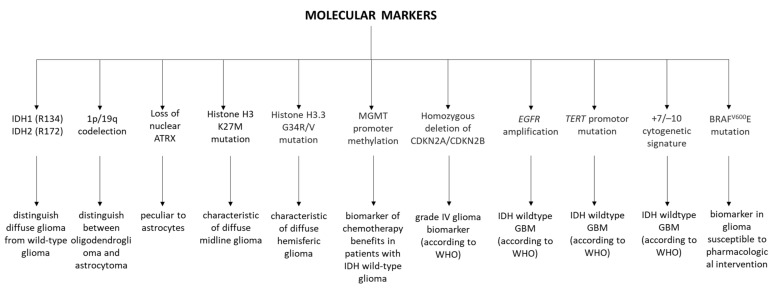
Diagram of molecular markers used in the diagnosis of brain gliomas. Diagram based on [1].

There are a number of molecular biomarkers used to categorize and distinguish diffuse gliomas, particularly in adults. These are shown in Figure 2.

Gliomas are histologically diverse. The most common are astrocytomas and glioblastomas. Less common are oligodendrogliomas, mixed oligodendrogliomas, and capillary astrocytomas. Many types of primary brain tumors show similar changes. Well-known and described pathways in glioblastoma biology rely on growth factor receptor tyrosine kinases that signal through the MAP cascade or PI3K signaling, and the loss of p53 apoptosis, cell cycle regulation, and VEGF-mediated angiogenesis. Specific changes in particular types of tumors were also identified, which were reflected in the specific behavior of certain molecular markers [6]. The characteristics of the changes in molecular markers depending on the neoplastic tumor are presented in Table 2.

Matrix metalloproteinases are responsible, in part, for the increased invasive capacity of gliomas, especially high-grade ones. MMP-1, also called interstitial collagenase, shows an especially significant overexpression of mRNA and proteins in astrocytomas characterized by a high degree of malignancy. It has also been shown that MMP-1 should not be present in a healthy brain. Nitric oxide (NO) is assumed to be associated with changes in expression. It has been shown that NO can directly affect the MMP-1 promoter, and therefore, the protein expression. High-grade tumors exhibit a significant excess of NO synthase, and it has been proven that in glioblastoma cells exposed to NO, there was a marked increase in MMP-1 expression [9].

Ubiquitination is a process consisting of a three-step enzymatic cascade in which proteins are labeled for degradation by a proteasome. UPS is involved in processes such as signal transduction, cell cycle regulation, transcription, and apoptosis. UPS in the brain became the subject of research after ubiquitin or UPS-related proteins were observed to be present in protein deposits in neurodegenerative diseases such as Alzheimer’s disease and Parkinson’s disease. As UPS plays a special role in many cellular functions, each mutation or abnormal expression of its components leads to all sorts of pathological conditions. These include cancer, neurodegenerative diseases, and immune disorders. In neoplastic diseases, ubiquitination is responsible for the activation or deactivation of the relevant oncogenic pathways [10].

Taking into account the mechanisms described above and the role of the studied proteins in their course, the hypothesis that MMP-1, UCHL-1, and the 20S proteasome are significantly involved in brain gliomas comes to mind. Changes in the expression of mRNA or proteins and changes in their amount in the brain, including the appearance of some of them, are the body’s response to the pathological condition leading to cancer. Each of the abnormalities has consequences in the form of an appropriate reaction stimulating an increase in the number of individual proteins, which should enable the assessment of the disease gradation. Therefore, this provides a kind of benefit as opposed to the study of only molecular markers, which are characteristic primarily of grade IV wild-type glioblastomas according to the WHO [1].

This study investigated three potential markers of brain gliomas. These are extracellular matrix metalloproteinase-1 (MMP-1), ubiquitin carboxy-terminal hydrolase L1 (UCH-L1), and the 20S proteasome.

MMP-1, as with other members of the metalloproteinase family, shows increased expression in many cancers. MMP-1 belongs to a family of zinc-dependent endopeptidases. It exhibits the ability to cleave the basic substrates of the extracellular matrix (ECM). The basic substrates for MMP-1 are collagen (types I, II, III, VII, and X), gelatinase, entactin, aggrecan, and tenascin. Despite general assumptions that MMP-1 should not be present in human brain tumors, mainly due to the lack of significant amounts of collagen in the brain structures, the level of MMP-1 in fact increases with an increase in the degree of malignancy of the disease and its invasiveness [11]. In the case of brain gliomas, an increase in MMP-1 expression is mainly observed in grade IV gliomas [12]. In addition to its well-known and described function of splitting the basic substrates of the extracellular matrix (ECM), MMP-1 also contributes to the activation of latent forms of bioactive molecules. As a result, it participates in the initiation of further pro-cancerogenic and pro-invasive signaling pathways [13].

UCH-L1 is a protein with a mass of 25 kDa. Under physiological conditions, it is expressed almost exclusively in the central and peripheral nervous systems. This protein has been linked primarily to the development of neurodegenerative diseases such as Alzheimer’s disease and Parkinson’s disease. An increased expression has also been demonstrated in neoplastic diseases, including cancer of the lungs and blood, as well as glioblastoma multiforme (GBM). Increased levels of UCH-L1 in the aforementioned tumors is correlated with a poor prognosis for the patient, as well as increased tumor invasiveness and metastatic disease behavior [14,15,16]. UCH-L1 is one of the deubiquitizing enzymes of the ubiquitin–proteasome system (UPS) [17].

A proteasome is a multi-protein complex that is responsible for the degradation of intracellular proteins [18]. Its primary function is to remove unnecessary regulatory proteins from the cell. The accumulation of these proteins would disrupt the cell’s functionality or cause its death [19]. This complex consists of, among others, the catalytic core to which the 20S proteasome belongs, and the regulatory particles making up the 26S proteasome. These particles are responsible for the identification, binding, and deubiquitination of the encountered substrates in the proteolytic chamber (20S proteasome) [18]. The proteasome in gliomas is probably responsible for the abnormal modulation of the brain tumor cell cycle. The inhibition of proteasome activity during treatment may bring more favorable results due to the ability to control the cell cycle [20].

Surface plasmon resonance (SPR), due to its high sensitivity to changes in the refractive index and the possibility of the label-free, sensitive, and quick detection of biomarkers, is a very good tool for biomedical diagnostics. It is an optical technique that can be successfully used for the quantification of biomarkers in biological fluids [21,22]. The essence of the SPR phenomenon is electron vibrations at the boundary of two phases—the conductive material and the dielectric material. This interaction causes the generation of a wave (the so-called surface plasmon wave). Its range covers the area between the layers of conductive and dielectric material, but does not extend more than 200 nm (in the perpendicular plane) [22].

The aim of this research was to develop a methodology for the diagnosis of a brain tumor by finding the interrelationships between MMP-1, UCH-L1, and the 20S proteasome, and by examining the influence of the degree of the glioblastoma, the patient’s age, and the tumor size on the amount of the measured proteins in the blood plasma.

In this study, we used biosensors previously constructed by our team based on the image version of the surface plasmon resonance technique for the quantitative determination of MMP-1, UCH-L1, and the 20S proteasome in the blood plasma of people with diagnosed glioblastomas and in a control group consisting of smokers of various ages. The results obtained were analyzed statistically. The resulting data set is one of the first to indicate the changes in the concentrations of the determined biomarkers depending on the grade of the disease and the patient’s general condition, as well as other potential carcinogenic factors.

## 2. Materials and Methods

### 2.1. Reagents

The following reagents were used for the tests: a standard solution of MMP-1 (Wuhan, China, USCN), rabbit anti-MMP-1 antibodies (RayBiotech, Inc., Peachtree Corners, GA, USA), recombinant human UCH-L1 and rabbit anti-UCH-L1 antibodies (R&D Sysytems, Minneapolis, MN, USA), 20S proteasome (AFFINITI Research Products Ltd., Exeter, UK), PSI (Z-Ile-Glu(OBut)-Ala-Leu-H) (BIOMOL, Hamburg, Germany), cysteamine hydrochloride, EDC [N-ethyl-N’-(3-dimethylaminopropyl) carbodiimide], NHS [N-hydroxysuccinimide] (Sigma Aldrich, St. Louis, MO, USA), HBS-ES solution (pH = 7.40, 0.01 M HEPES, 0.15 M sodium chloride, 0.005% Tween-20, 3 mM EDTA), and PBS (pH = 7.40, phosphate-buffered saline) (BIOMED).

### 2.2. Biological Material

Tests were performed on the blood plasma of people diagnosed with G1 to G4 brain gliomas, and on the blood plasma of smokers as a control group (K). A total of 105 plasma samples were tested, including 48 from the control group. The biological material was obtained from the Biobank of the Medical University in Bialystok, and the relevant consent from the bioethics committee was obtained for the research (license APK.002.171.2021). Detailed data on the research material collected from the group of patients are given in Table A1 in Appendix A.

### 2.3. Procedure for Quantifying MMP-1, UCH-L1, and Proteasome—SPRi Measurements

**Figure A1:**
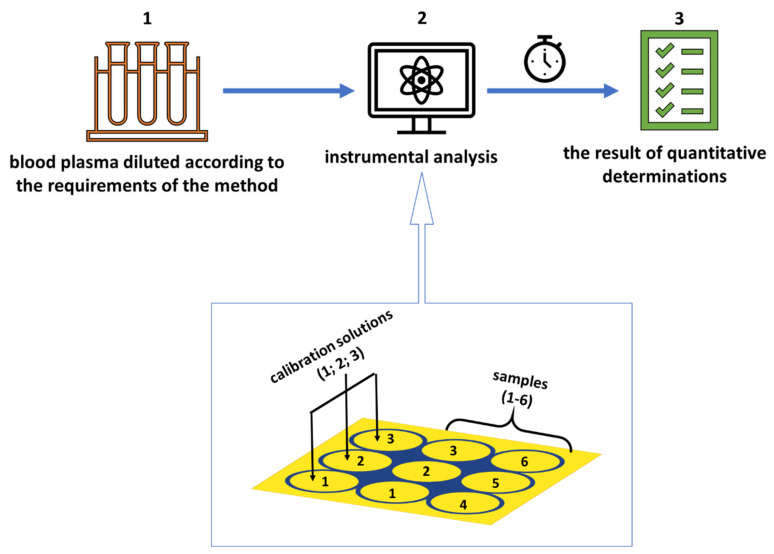
Scheme of analytical procedure for quantification of MMP-1, UCH-L1, and 20S proteasome in blood plasma.

Quantitative determinations of the biomarkers selected for the study were made with the use of SPRi biosensors. The most important parameters of the methods used are given in Table 3. Initially, the blood plasma was diluted (in PBS buffer) so as to fit the analytical signal to the linear range of the calibration curve. Next, an instrumental analysis was performed. The first step was to coat the surface-functionalized biosensor plate with cysteamine with a ligand (receptor), an element that captures the analyte from the solution. For this purpose, standard solutions of the appropriate ligand were applied to the active sites of the biosensor (each had nine such sites), and then the biosensor plate was placed in an incubator at 37 °C for one hour. After this time, the surface of the biosensor was washed with distilled water and HBS-ES buffer solution. The biosensor was placed on a prism in the SPRi device, and a series of photographs were taken over the selected optimal range of SPR angles. The following were then applied to the biosensor plate: three standard solutions of the tested protein, which were used for each calibration, and six appropriately diluted blood plasma samples. The ligand–analyte interaction time was 10 min. After this time, the surface of the biosensor was washed with distilled water and HBS-ES solution, and again a series of photographs were taken in the same range of angles as before. Finally, the analysis was performed at only one value of the SPR angle: that giving the greatest difference in the light intensity of the biosensor active site before and after the interaction with the analyte. ImageJ software (NIH, version 1.8.0_172) was used for the mathematical processing of the obtained images. A flowchart of the analytical procedure is presented in Figure A1 in Appendix A.

### 2.4. IDH 1/2 Mutation, p53 Gene Mutation, and EGFR Expression

Data on the occurrence of mutations or expression of molecular markers were taken from the available medical documentation describing the tested samples.

## 3. Results

### 3.1. Statistical Analysis

Statistical analyses were performed using PQStat Software (2022), PQStat v.1.8.4, Poznan, Poland.

**Figure 3:**
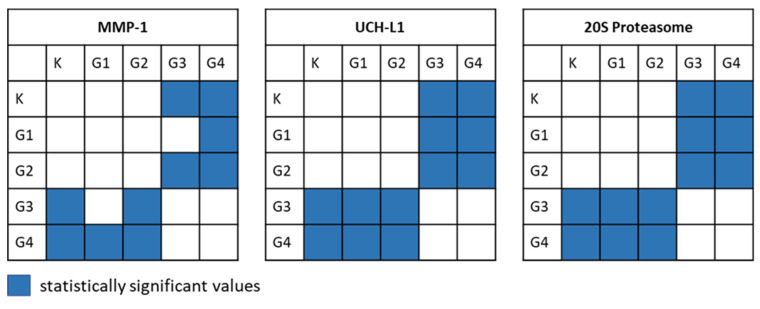
Graphical representation of the Dunn–Bonferroni post hoc test results.

To check the normality of the distribution of the results, the Shapiro–Wilk test was performed. The result indicated the lack of normality of the distribution of the analyzed data. This necessitated the use of nonparametric statistical tests. The first of these was the Kruskal–Wallis test, used to test the hypothesis (H_0_) that there were no statistically significant differences between the median concentrations in the groups from K to G4. Because this test showed a relationship *p* << α for each of the quantified proteins, H_0_ was rejected in favor of the alternative hypothesis (H_1_) that not all medians were equal. The Dunn–Bonferroni post hoc test was performed to determine which data pairs were significant. The results of this analysis are presented graphically in Figure 3.

The analysis shows that there are statistically significant differences in the median concentrations of individual proteins between the groups K and G3 and between K and G4, and also between G1 and G3 (except in the case of MMP-1) and G1 and G4. A similar relationship was also noted for groups G2 and G3 and for G2 and G4.

**Figure 4:**
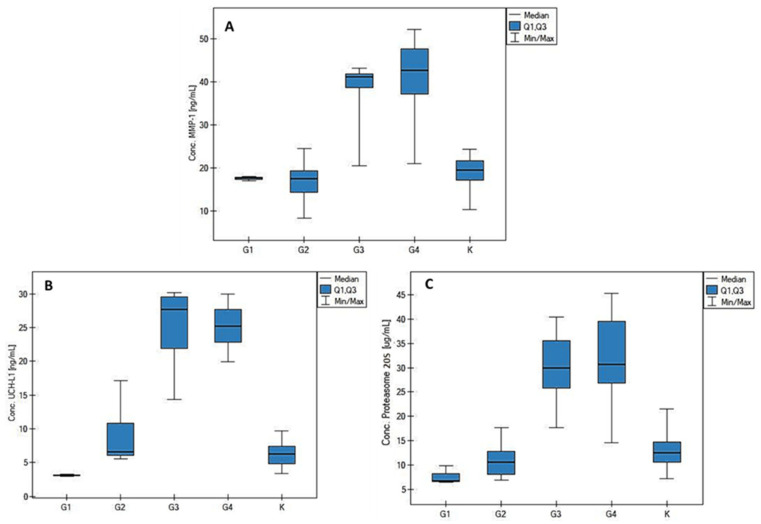
Graphs of changes in the concentrations of (**A**) MMP-1, (**B**) UCH-L1, and (**C**) 20S proteasome depending on the grade of the disease and in the control group.

Figure 4 shows the changes in the concentrations of the tested potential biomarkers of brain gliomas depending on the grade of the disease and in the control group. Detailed values of Q1, Q3, and the median and breakpoints of individual concentrations for each protein and grade are summarized in Table A2 in Appendix A.

Table A3 in Appendix A presents the detailed relationships between the concentrations of individual proteins and the grade of the disease, the size of the neoplastic tumor, the presence of neoplasms other than glioblastomas in the patient’s immediate family, and the presence of other non-neoplastic diseases in the studied patient. The data show that if the grades G1–G2 are compared with G3–G4, then for each of the potential biomarkers there is a statistically significant difference in its median concentration (*p* << 0.01). Unfortunately, this was the only relationship identified in this analysis; the size of the neoplastic tumor, tumors in the patient’s family, and coexisting diseases other than neoplasms did not statistically significantly affect the concentrations of MMP-1, UCHL-1, or the 20S proteasome (*p* > 0.05 for each protein and analyzed parameter).

Correlations between the patient’s age and the protein concentrations, the size of the tumor and the protein concentrations, and between different proteins were investigated using nonparametric tests. For this purpose, Spearman’s rank correlation (R_S_) was used, with a classification based on the scale proposed by J. Guilford. By comparing the patient’s age and the concentration of individual proteins (without division into disease grades), we obtained results in the range of 0.3 < R_S_ ≤ 0.5 (*p* < 0.05), indicating a positive average correlation. No relationship was found when analyzing the tumor size in relation to the concentration of each protein; in each case, the result was not statistically significant (*p* > 0.05). When analyzing the correlation between proteins, with no division of samples into grades of the disease, we obtained correlation results in the range of 0.5 < R_S_ ≤ 0.7 (*p* << 0.01), which allowed this relationship to be classified as a positive high correlation. Detailed data on the correlation results can be found in Table A4 and Table A5 in Appendix A.

### 3.2. ROC Analysis

**Figure 5:**
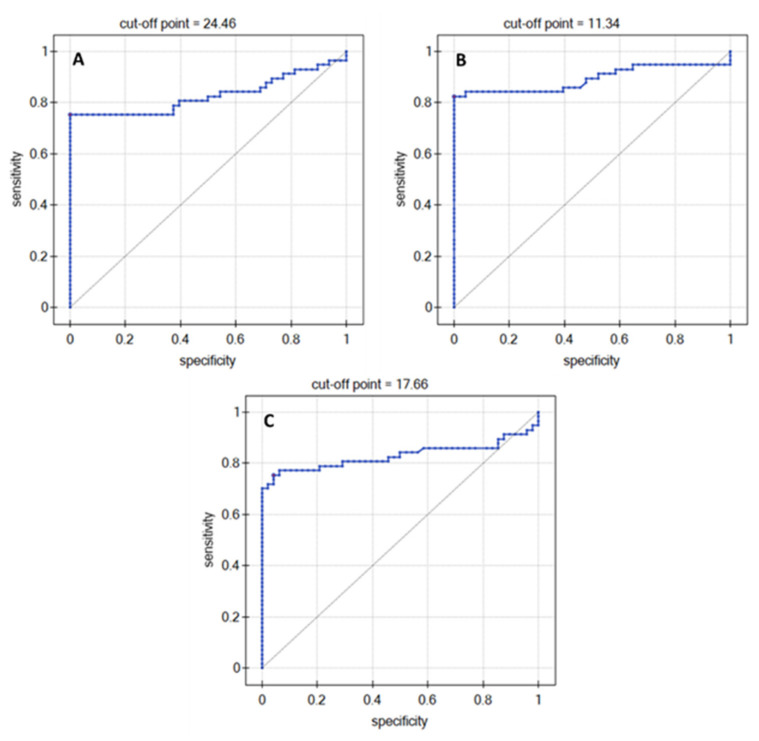
ROC curves: (**A**) MMP-1, (**B**) UCH-L1, and (**C**) 20S proteasome.

An ROC analysis was performed to determine whether it was possible to distinguish the control group from patients with a diagnosed glioblastoma. The obtained data set suggested that it is possible only to distinguish milder grades of the disease and the control group (K/G1/G2) from the more advanced forms (G3/G4). This conclusion was based on the cut-off point on the ROC curve. The value of this point and the course of the ROC curves are presented in Figure 5. Table 4 contains data illustrating the diagnostic efficacy of MMP-1, UCH-L1, and the 20S proteasome in plasma for brain gliomas.

### 3.3. Impact of IDH Status on Biomarkers

**Figure 6:**
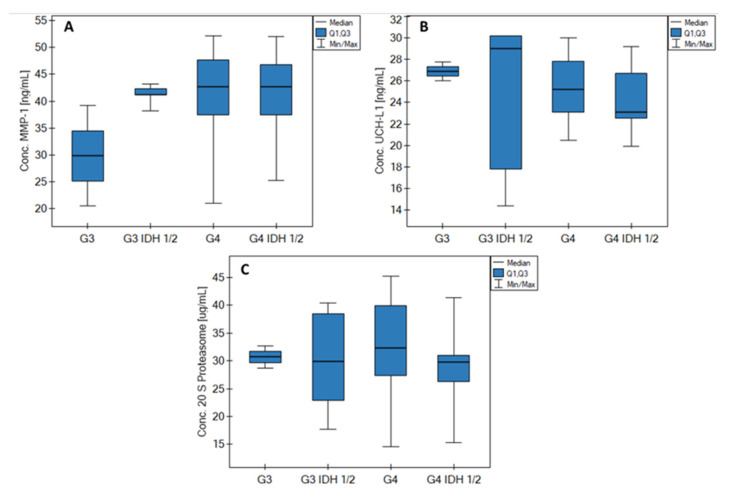
Impact of IDH status on biomarkers (A) MMP-1; (B) UCH-L1 and (C) 20S proteasome.

The effect of the IDH 1/2 status on the measured biomarkers was examined. Statistical analyses were performed in the form of the Kruskal–Wallis test and the Dunn–Bonferroni post hoc test. The results of the statistical analyses indicated no statistically significant differences between the median concentrations of individual proteins in relation to the samples showing the IDH 1/2 mutation to those that did not. The post hoc test (Dunn–Bonferroni) also did not indicate any group combinations that could show statistically significant differences. Thus, the IDH 1/2 status was not found to have an effect on the biomarkers investigated in this study. The size of the analyzed samples probably had a certain impact, amounting to: G3 (2), G3 IDH 1/2 (5), G4 (27), and G4 IDH 1/2 (10). Nevertheless, we cautiously drew the preliminary conclusion that none of the measured biomarkers (MMP-1, UCHL-1, or the 20S proteasome), or more specifically their concentrations, depended in any way on the IDH 1/2 status. The obtained results are shown in Figure 6.

## 4. Discussion and Conclusions

Techniques and diagnostic methods for the efficient diagnosis of neoplastic diseases are constantly being modified and updated. The present work is an attempt to modify and propose another diagnostic technique, based on the quantification of MMP-1, UCH-L1, and the 20S proteasome in the blood plasma. The results of such quantification for people with brain glioblastomas were compared with the concentrations of those proteins in the blood plasma of the control group. The SPRi biosensors used represent a class of liquid biopsy methods. These biosensors provide a fast, inexpensive, and environmentally friendly chemical analysis of biological material. A total of 105 blood plasma samples were tested for their content of MMP-1, UCH-L1, and the 20S proteasome, and the results were analyzed statistically. There were statistically significant differences in the protein concentrations between K and G3/G4, and also between G1 and G3/G4, except that in the case of the MMP-1 concentration, no difference was found between the G1 and G3 groups. A similar relationship was also noted for grades G2 and G3 and for G2 and G4.

A disadvantage of the proposed diagnostic method is that the concentrations of each of the analyzed proteins in groups K, G1, and G2 remained at a similar level, which disqualifies this method from being used for screening in cases where there is no suspicion of a likely brain glioma. A noticeable increase in concentrations was seen in the G3/G4 grades, and the concentrations of individual proteins also remained at a similar level between these two grades. This can be explained by the fact that the tests were carried out on blood plasma, and the blood–brain barrier (BBB) was probably not sufficiently damaged to enable the proteins being measured to cross it freely and enter the bloodstream [26]. The BBB provides a physical and biochemical barrier to paracellular diffusion, which means that molecules in the bloodstream can only be transported to the brain through appropriate cell membranes. The BBB also restricts the proper distribution of the oncological drugs used, which is why brain gliomas are extremely difficult to cure with the use of oncological chemistry. However, abnormalities in the brain, including cancerous tumors, can disrupt the BBB by disrupting its integrity and allowing molecules from the brain to enter the bloodstream and vice versa. Most often, BBB disruption is observed in GBM, although it is not a rule, because not all BBB regions in GBM are physically damaged, which can be observed using magnetic resonance imaging [27]. In addition, brain tumors have a unique ability to secrete some components of the extracellular matrix (ECM), including collagens responsible for the rigid and continuous structure of the BBB [28].

The difference in concentrations between the grades G1/G2 and G3/G4 exhibited a statistical significance. Therefore, the proposed methods may provide an extension of the diagnostic methods currently used, serving to confirm or exclude the presence of a mild or highly invasive brain glioma.

Probably all these features, both of the BBB tumor and the brain tumor itself, effectively prevent the early diagnosis of gliomas using the proteins analyzed in this article. The proposed biomarkers are unlikely to completely replace histological and imaging studies, but it is worth noting that thanks to the marked changes in the concentrations between the G1/G2 and G3/G4 stages, they offer the possibility of at least a preliminary assessment, in conjunction with other diagnostic methods used, of the stage of the brain tumor and tumor progression in higher grades.

Using Spearman’s rank correlations, an analysis was performed on the correlations between the parameters available in the clinical description and the concentrations of individual proteins. The division into disease grades did not yield any correlation results. Significant results were obtained only when analyzing all samples simultaneously. The concentrations of the individual proteins showed a positive average correlation with the patient’s age. However, no relationship was found between the size of the tumor and the concentration of any potential biomarker. A positive high correlation was determined between the concentrations of the particular proteins.

The ROC analysis confirmed the earlier observations. It indicated that it is possible to distinguish K and G1/G2 from the G3/G4 grades.

**Figure A2:**
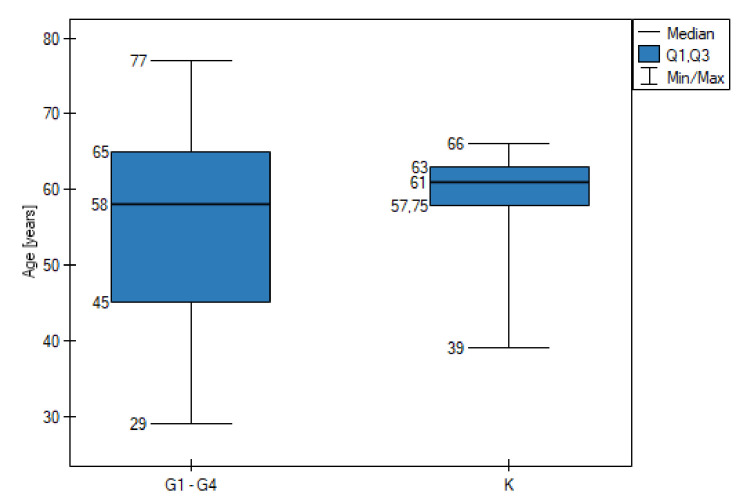
Age comparison of the patient population and the control group.

**Figure A3:**
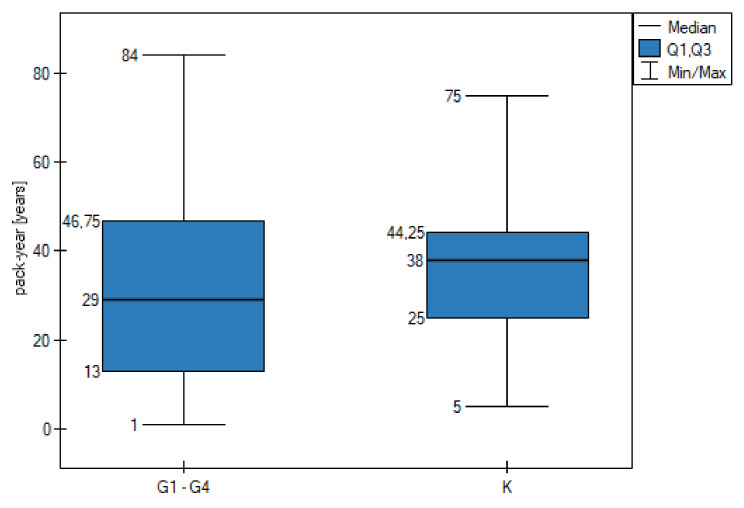
Comparison of the patient population and the control group in relation to pack-years.

Age was summarized according to the severity of the brain glioma and in the control group. It was noticed that the median age increased with the grade of the disease and amounts to: G1 (39 years), G2 (43.5 years), G3 (45 years), G4 (60 years), and K (61 years), respectively. It was also checked whether there was a difference between the medians of age in the population of patients and the control group. The results of the statistical analysis contradicted the supposition that there were statistically significant differences in these two populations. The data are illustrated in Figure A2 in Appendix A. Taking into account gender, the control group consisted of almost an equal number of women and men, while a clear difference was observed in the case of grade G4 (26 men vs. 11 women), which may suggest that men suffer from gliomas more often. When we looked at the presence of neoplasms other than glioblastomas in the patient’s immediate family, we noticed that the group in which the neoplasms were present and the group in which they were not found were almost equal, so this parameter cannot have a real impact on the risk of developing a brain glioma. The situation is similar in the case of other comorbidities in relation to the diagnosed patient. Moreover, in the vast majority of cases, the astrocyte type of neoplastic tumor predominated. The occurrence of mutations and the overexpression of molecular markers such as IDH 1/2, p53, and EGFR were also summarized. In most cases, mutations (IDH 1/2, p53) or over-expressions (EGFR) were present in the diagnosed patients. The exception was the IDH 1/2 mutation in the G4 grade, where in 27 (*n* = 37) cases, no mutation was observed. The analysis of the influence of the IDH 1/2 mutation, p53 mutation, and EGFR overexpression on the concentrations of the measured proteins did not show that the concentrations of MMP-1, UCH-L1, and the 20S proteasome depended on the mentioned molecular markers. It was also analyzed whether the control group (heavy smokers) was not too biased. For this purpose, Table A1 in Appendix A shows the number of cases of heavy smokers in the group of patients and the median pack-years (both in G1–G4 and K) that were drawn. A relevant statistical analysis was performed, the results of which contradicted the bias of the control group. The data are illustrated in Figure A3 in Appendix A.

Our results confirmed the findings of previous studies [11,12] regarding an increase in the MMP-1 concentration with an increase in the degree of invasiveness of the disease, despite the lack of significant amounts of collagen in the brain. The situation was similar for the level of UCH-L1. For this protein, a more advanced grade and increased invasiveness of the disease also resulted in higher levels, as has been reported in previous studies [14,15,16]. In brain tumors, the 20S proteasome is responsible for abnormalities in the tumor cell cycle, causing a loss of control of life processes in the cell [20]. It would seem logical that an increase in the invasiveness of the tumor would lead to an increased amount of the 20S proteasome. In this study, the largest increase in the 20S proteasome concentration was observed for grades G3 and G4.

In most cases, the authors of the cited works studied the expression of particular proteins in question. The exception to this is UCH-L1 in one of the articles, which presented the exact concentration of the protein in the cerebrospinal fluid in Alzheimer’s disease [16]. The others were based on changes in the expression of MMP-1, UCH-L1, and the 20S proteasome in various diseases, not only in glioblastomas. They concerned changes in gynecological, lung, and hematological cancers, as well as Parkinson’s disease. Our team provided the first data on the concentrations of MMP-1, UCH-L1, and the 20S proteasome and their interrelationships in blood plasma. Using the concentration values, we also correlated the amount of a given protein depending on the patient’s age and the size of the neoplastic tumor. The concentration values were also used to determine the diagnostic values of MMP-1, UCH-L1, and the 20S proteasome by plotting ROC curves for brain glioma.

Proteasome inhibitors also play a role in brain gliomas. Many clinically approved proteasome inhibitors have been shown to be capable of inhibiting GBM in both genetic and pharmacological studies. Proteasome inhibitors trigger anti-tumor activity by disrupting the balance and accumulation of proteins, resulting in the disruption of signaling pathways and the activation of apoptosis. Protein synthesis and autophagy are unique, so-called metabolic signatures that are useful for identifying GBMs susceptible to proteasome inhibition. UPS and autophagy are responsible for the control of cellular fitness, so directing treatment towards proteomic imbalance in GBM through the use of proteasome inhibitors may be a novel therapeutic route for cancer [29].

The proposed analytical method is important as an additional study accompanying imaging diagnostics, especially in the differentiation of G2 and G3 gliomas. There are indications that it is difficult to distinguish between the two cases based solely on imaging diagnostics. The proposed biomarkers could be particularly useful for this type of research, due to the demonstrated statistically significant difference between the G2 and G3 grades in the conducted research.

## Figures and Tables

**Table 1:** Preoperative methods and diagnostic techniques used for the diagnosis of brain gliomas.

Preoperative Diagnostic Method/Technique	Application	Ref.
MRI of the brain with various modifications (including the use of a gadolinium-based contrast agent)	The gold standard in the diagnosis of brain gliomas.	[2]
Perfusion MRI and PET	Determination of metabolic hotspots for tumor tissue harvesting when a biopsy is considered, rather than open resection.	[3]
Electroencephalography	Monitoring of epilepsy as the cause of the neoplastic tumor; helps in determining the cause of consciousness disorders.	[4]
Liquid biopsy	Detection of cell-free DNA of a brain tumor; liquid biopsy methods for screening, early diagnosis, and pre-operative diagnosis require further development to confirm their effectiveness.	[4]

**Table 2:** Characteristics of selected molecular markers of brain gliomas.

Marker	Histological Type of Tumor	Characteristics of Marker Changes	Ref.
BRAF	Pilocytic astrocytoma	Mutations or fusions in the BRAF gene.	[6]
IDH1 CIC FUBP1	Oligodendroglioma	The IDH1 mutation and the 1p19q chromosome co-deletion correlate with higher survival during the administration of combined chemotherapy and radiotherapy. Moreover, oligodendrogliomas are characterized by unique mutations of the CIC and FUBP1 genes.	
IDH p53	Astrocytoma, primary glioblastoma	The development of astrocytomas through the IDH mutation followed by p53. EGFR amplification and loss of PTEN and cyclin-dependent kinase inhibitors in primary glioblastoma.	
IDH 1p/19q MGMT	Diffuse gliomas	IDH mutation, 1p/19q co-deletion, and MGMT methylation as prognostic markers of diffuse gliomas.	
MGMT promoter methylation	Anaplastic glioma, glioblastoma multiforme (GBM)	MGMT promoter methylation is a prognostic marker for low-grade gliomas (about 93%), anaplastic gliomas (50–80%), primary glioblastomas (about 40%), and secondary glioblastomas (about 70%).	[7]
ATRX	Secondary glioblastoma, low-grade glioma	Mutation or deletion of the ATRX gene, which is also found in IDH mutant tumors that are not 1p/19q co-deleted.	[8]
EGFR	Primary glioblastoma	Amplification, mutation, and mutual exclusion of p53 mutations. EGFR amplification occurs in approximately 40% of GBM patients and correlates with high-grade neoplasm.	
HIF-1α	High-grade glioma	Overexpression.	
NF1	Mesenchymal and pilocytic astrocytomas Primary glioblastoma	About 20% mutation or deletion in the primary glioblastoma. Close association of NF1 with the mesenchymal subtype.	
VEGF	Mesenchymal glioma	Overexpression correlates with a higher grade of glioma.	
ELDT1	Mesenchymal and high-grade gliomas	Overexpression.	

**Table 3:** Characteristics of analytical methods and procedures used to quantify the concentration of the tested proteins.

Analytical Characteristics of the Methods Used				Characteristics of the Quantification Procedures Used		
Biomarker	Method	Analytical Characteristic	Ref.	Biological Material	Dilution	pH
MMP-1	SPRi biosensor (non-fluidic)	Cantibody = 170 ng/mL	[23]	**Blood plasma** (blood centrifuged at 3000 rpm for 15 min, then stored at −80 °C)	K	7.40
		LOD = 9 pg/mL			G1 ×15	
		LOQ = 18 pq/mL			G2	
		pH = 7.40			G3 ×30	
		LR: 0.1–2.5 ng/mL			G4	
UCH-L1		Cantibody = 10 µg/mL	[24]		K	
		LOD = 0.06 ng/mL			G1 ×10	
		LOQ = 0.19 ng/mL			G2	
		pH = 7.40			G3 ×15	
		LR: 0.5–3.0 ng/mL			G4	
20S Proteasome		CPSI = 25 µg/mL	[25]		K	
		LOD = 0.1 µg/mL			G1 ×5	
		LOQ = 0.37 µg/mL			G2	
		pH = 7.40			G3 ×10	
		LR: 1.4–7.0 µg/mL			G4	

LR—linear range of calibration curve.

**Table 4:** Data on the diagnostic performance of the tested proteins.

	AUC	*p*-Value	PPV (%)	NPV (%)	Sensitivity (%)	Specificity (%)	Cut-Off Point
MMP-1	0.83	<<0.01	100	77.42	75.43	100	24.46
UCH-L1	0.89	<<0.01	100	82.76	82.46	100	11.34
20S Proteasome	0.83	<<0.01	95.56	76.67	75.44	95.83	17.66

## Data Availability

Not applicable.

## Appendix A

**Table A1:** Clinicopathological characteristics of patients.

Control Group/Tumor Grade							
Variable		K *n* = 48	G1 *n* = 3	G2 *n* = 10	G3 *n* = 7	G4 *n* = 37	Age Difference Between K and G1–G4 ^A^
Age (years)	range	39–66	29–43	30–57	38–72	33–77	No statistically significant difference (*p* > 0.05)
	median	61	39	43.5	45	60	
Gender	male	27	1	5	3	26	
	female	21	2	5	4	11	
Tumor size (cm^2^)	<15		2	2	3	10	
	>15		1	2	1	10	
The presence of other neo-plasms in the immediate family	yes		0	6	3	17	
	no		3	4	4	20	
Concomitant non-cancerous diseases	yes		2	3	3	21	
	no		1	7	4	16	
Histological type of tumors	oligodendroglial		0	1	3	0	
	astrocytic		3	9	4	37	
Number of smokers	number of cases	48	1	6	4	22	Pack-year difference between K and G1–G4 ^B^
	pack-years (median)	37.5	0 ^C^	8	26	38.5	No statistically significant difference (*p* > 0.05)
IDH 1/2 mutation	yes		0	10	5	10	
	no		3	0	2	27	
p53 mutation	yes		1	10	7	34	
	no		2	0	0	3	
EGFR expression	yes		0	6	2	29	
	no		3	4	5	8	

^A B^ Mann–Whitney U test; ^C^ one case was 21 pack-years.

**Table A2:** Detailed data for Figure 4.

Parameter/Biomarker	MMP-1 (ng/mL)					UCH-L1 (ng/mL)					Proteasome (µg/mL)				
	K	G1	G2	G3	G4	K	G1	G2	G3	G4	K	G1	G2	G3	G4
**Median**	19.43	17.68	17.49	41.22	42.73	6.24	3.08	6.57	27.73	25.16	12.59	6.81	10.67	29.87	30.73
**Q1**	17.22	17.33	14.28	38.69	37.16	4.83	3.03	6.06	21.89	22.83	10.66	6.66	8.08	25.81	26.77
**Q3**	21.70	17.82	19.34	41.77	47.65	7.42	3.20	10.82	29.55	27.67	14.76	8.35	12.91	35.57	29.49
**MIN**	10.24	16.98	8.32	20.55	21.06	3.43	2.97	5.54	14.39	19.89	7.19	6.51	6.90	17.66	14.56
**MAX**	24.40	17.96	24.46	43.23	52.15	9.66	3.31	17.15	30.17	29.98	21.52	9.89	17.68	40.42	45.25

**Table A3:** Changes in MMP-1, UCH-L1, and 20S proteasome concentrations in comparison with the available clinical data of the samples from the patients.

Parameter	MMP-1 *Concentration (ng/mL)*			UCH-L1 *Concentration (ng/mL)*			20S Proteasome *Concentration (µg/mL)*		
	Range	Median	*p*-Value	Range	Median	*p*-Value	Range	Median	*p*-Value
Tumor grade (G1–G2 vs. G3–G4)									
G1 (3)	16.98–17.96	17.68	<<0.01	2.97–3.31	3.08	<<0.01	6.51–9.89	6.81	<<0.01
G2 (10)	8.32–24.46	17.49		5.54–17.15	6.56		6.90–17.68	10.67	
G3 (7)	20.55–43.23	41.22		14.39–30.17	27.73		17.66–40.42	29.87	
G4 (37)	21.06–52.15	42.73		19.89–29.98	25.16		14.56–45.25	30.73	
Tumor size (cm^2^)									
<15 (17)	8.32–47.78	36.73	>0.05 (NS)	2.97–30.14	23.03	>0.05 (NS)	6.81–44.28	28.74	>0.05 (NS)
>15 (14)	13.97–51.98	42.32		3.08–29.38	23.24		6.51–39.78	30.50	
The presence of other neoplasms in the family									
yes (26)	8.32–52.15	39.67	>0.05 (NS)	6.31–30.17	22.51	>0.05 (NS)	7.21–45.25	27.92	>0.05 (NS)
no (29)	9.03–51.98	38.43		2.97–30.14	24.02		6.51–44.28	28.34	
Concomitant non-cancerous diseases									
yes (29)	9.03–51.23	40.46	>0.05 (NS)	2.97–29.03	23.21	>0.05 (NS)	6.51–45.25	28.34	>0.05 (NS)
no (28)	8.32–52.15	38.19		3.31–30.17	24.07		6.90–44.77	27.92	

(NS)—no statistically significant values.

**Table A4:** Correlations: protein concentration–patient’s age and protein concentration–tumor size.

**Age–MMP-1**					
	**G1**	**G2**	**G3**	**G4**	**All Samples**
**R_Spearman_**	0.5	−0.59	−0.02	−0.18	0.34
***p***	>0.05	>0.05	>0.05	>0.05	<0.01
**Age–UCH-L1**					
	**G1**	**G2**	**G3**	**G4**	**All Samples**
**R_Spearman_**	0.5	−0.26	−0.29	−0.04	0.31
***p***	>0.05	>0.05	>0.05	>0.05	<0.05
**Age–20S Proteasome**					
	**G1**	**G2**	**G3**	**G4**	**All Samples**
**R_Spearman_**	1	0.13	0.31	0.05	0.48
***p***	-	>0.05	>0.05	>0.05	<0.01
**Tumor Size–MMP-1**					
	**G1**	**G2**	**G3**	**G4**	**All Samples**
**R_Spearman_**	-	-	-0.4	0.36	0.24
***p***	-	-	>0.05	>0.05	>0.05
**Tumor Size–UCH-L1**					
	**G1**	**G2**	**G3**	**G4**	**All Samples**
**R_Spearman_**	-	0.4	-	0.09	−0.11
***p***	-	>0.05	-	>0.05	>0.05
**Tumor Size–20S Proteasome**					
	**G1**	**G2**	**G3**	**G4**	**All Samples**
**R_Spearman_**	-	-	0.8	0.08	0.04
***p***	-	-	>0.05	>0.05	>0.05

**Table A5:** Correlation analysis between proteins.

**G1**			
	**MMP-1–UCH-L1**	**MMP-1–Proteasome**	**UCH-L1–Proteasome**
**R_Spearman_**	-	0.5	0.5
***p***	-	>0.05	>0.05
**G2**			
	**MMP-1–UCH-L1**	**MMP-1–Proteasome**	**UCH-L1–Proteasome**
**R_Spearman_**	0.07	0.07	0.14
***p***	>0.05	>0.05	>0.05
**G3**			
	**MMP-1–UCH-L1**	**MMP-1–Proteasome**	**UCH-L1–Proteasome**
**R_Spearman_**	0.32	-	0.25
***p***	>0.05	>0.05	>0.05
**G4**			
	**MMP-1–UCH-L1**	**MMP-1–Proteasome**	**UCH-L1–Proteasome**
**R_Spearman_**	0.32	0.16	0.21
***p***	>0.05	>0.05	>0.05
**All samples**			
	**MMP-1–UCH-L1**	**MMP-1–Proteasome**	**UCH-L1–Proteasome**
**R_Spearman_**	0.63	0.59	0.61
***p***	<<0.01	<<0.01	<<0.01
